# Prognostic Value of Bone Metastases by Extent of Disease and Lung Metastases in High-Volume Castration-Sensitive Prostate Cancer: A Retrospective Study

**DOI:** 10.3390/cancers17203306

**Published:** 2025-10-13

**Authors:** Dai Koguchi, Hideyasu Tsumura, Ken-ichi Tabata, Shuhei Hirano, Soichiro Shimura, Takefumi Satoh, Masaomi Ikeda, Daisuke Ishii, Kazumasa Matsumoto

**Affiliations:** 1 Department of Urology, Kitasato University School of Medicine, 1-15-1 Kitasato, Minami-ku Sagamihara, Kanagawa 252-0374, Japankazumasa@cd5.so-net.ne.jp (K.M.); 2 Department of Urology, Kitasato Institute Hospital, 5-9-1 Shirokane, Minato-ku, Tokyo 108-8642, Japan

**Keywords:** bone metastasis, lung metastasis, prostate cancer, CHAARTED criteria

## Abstract

**Simple Summary:**

In this study, we conducted a retrospective assessment of the metastatic patterns associated with a favorable prognosis in metastatic castration-sensitive prostate cancer with bone metastasis, including the coexistence of lung metastasis. Our multivariate analyses of overall survival and castration resistance-free survival revealed that prognoses worsened as the extent of bone metastases increased. However, the coexistence of lung and bone metastases had no prognostic impact. We conclude that prostate cancer with fewer bone metastases, even with lung metastasis, should be viewed differently from the prevalent notion that metastatic prostate cancer generally has an unfavorable prognosis.

**Abstract:**

Backgrounds: High-volume (HV) metastatic castration-sensitive prostate cancer (mCSPC) is an aggressive disease. Despite this, we aimed to assess the metastatic patterns associated with a favorable prognosis in HV disease with bone metastasis (BM), including BM’s coexistence with lung metastasis (LM). Methods: We retrospectively analyzed 379 patients with synchronous mCSPC. They were categorized using the CHAARTED criteria as low-volume (LV) or HV with BM, classified based on extent of the disease from 1 to 4 (HV-EOD1–4) with or without LM. Multivariate Cox models for overall survival and castration-resistance-free survival assessed the prognostic values of HV-EOD1–4 compared with LV disease and the presence of LM. Site-specific radiographic progression at the time of castration-resistant prostate diagnosis was assessed in patients with BM and LM. Results: Multivariate analyses for overall survival showed no prognostic value of HV-EOD1 (hazard ratio [HR] 0.90; 95% confidence interval [CI] 0.43–1.85; *p* = 0.77), HV-EOD2 (HR 1.17; 95% CI 0.69–1.99; *p* = 0.57), and LM (HR 1.29; 95% CI 0.80–2.07; *p* = 0.29). In the analyses, HV-EOD ≤ 2 and LM did not influence castration resistance-free survival. LM showed a significantly lower incidence of radiographic progression to castration-resistant prostate cancer than BM (6.0% vs. 29.9%, *p* < 0.001). Conclusions: This study indicates the prognostic heterogeneity of HV disease considering BM and LM. These findings may aid in determining the treatment intensity for mCSPC.

## 1. Introduction

Most guidelines use the CHAARTED criteria as a diagnostic tool to evaluate tumor aggressiveness in metastatic castration-sensitive prostate cancer (mCSPC) [1,2,3]. High-volume (HV) disease, defined using the CHAARTED criteria, generally indicates aggressive behavior. In the CHAARTED trial, patients with HV disease had a median overall survival (OS) of approximately 2 years less than those with low-volume (LV) disease in the androgen deprivation therapy (ADT) alone group [4].

Two recent network meta-analyses of HV disease suggest a reasonable association between using more therapeutic agents and improved prognosis. Specifically, adding docetaxel to an androgen receptor signaling inhibitor plus ADT was more effective than using an androgen receptor signaling inhibitor plus ADT [5,6]. However, docetaxel may lead to serious or persistent adverse events, such as neutropenic fever, interstitial pneumonia, and peripheral neuropathy, which decrease the quality of life, especially in patients over 70 years who comprise the majority of patients with mCSPC [7,8,9,10]. Therefore, the optimal treatment intensity for HV disease remains undetermined.

The optimal treatment strategy for HV disease can be identified through subcategorization. HV disease is characterized by the presence of visceral metastases (VM) or four or more bone metastases (BM) with at least one of those locations outside the vertebral body or pelvis [4,11]. Thus, HV disease comprises a wide spectrum, including extensive BM and VM with or without BM. Therefore, we hypothesized that subcategorizing this heterogeneous disease would help identify metastatic patterns with only mildly aggressive behavior, guiding decisions regarding treatment intensity in patients with mCSPC during the current upfront era.

In this study, we aimed to assess the prognostic value of HV disease, in relation to bone metastatic burden, compared to LV disease. Additionally, we examined the prognostic value of coexisting lung metastasis (LM) in patients with mCSPC and BM.

## 2. Materials and Methods

### 2.1. Study Population

After the Institutional Review Boards of Kitasato University School of Medicine and Kitasato University Hospital (Kanagawa, Japan) (approval numbers B23-106, B22-102, and B24-120) approved this study, we retrospectively reviewed the clinical data of 451 patients diagnosed with synchronous mCSPC at Kitasato University Hospital between April 2006 and September 2023. This study was conducted under the Declaration of Helsinki and its contemporary (2013) amendments. The requirement for informed consent was waived by the Institutional Review Board owing to the retrospective nature of this observational study.

Among the 145 patients with LV disease and 306 with HV disease according to the CHAARTED criteria, determined by computed tomography and technetium-99 bone scanning, we focused on the data from patients with BM with and without LM, regardless of distal lymph node metastasis. Patients with LV disease with EOD2 or at least one VM that was not LM, such as the liver, adrenal gland, or mediastinum, were excluded from the study. The initial diagnosis of prostate cancer (PCa) was histologically confirmed in all cases.

Data regarding patient characteristics were obtained from medical records. They included demographic data, the Charlson Comorbidity Index [12], disease volume classified as HV or LV, extent of disease (EOD) classified by bone scintigraphy [11], and prognostic variables, including radiographic progression (RP), castration resistance-free survival (CRFS), and OS. All patients initially received ADT comprising luteinizing hormone-releasing agonists/antagonists or a combined androgen blockade (CAB) with ADT plus a nonsteroidal antiandrogen. Intensified ADT for mCSPC involves doublet therapy with ADT plus abiraterone, enzalutamide, apalutamide, or docetaxel.

### 2.2. Definitions of Endpoints

The primary endpoint was OS, characterized by the interval from the date treatment for mCSPC began to the date of death from any cause. The secondary endpoint was CRFS, defined as prostate-specific antigen (PSA) failure or RP following the Prostate Cancer Working Group 2 or 3 criteria [13,14].

### 2.3. Statistical Analysis

HV disease with BM was classified as EOD from 1 to 4 (HV-EOD1–4). Also, OS and CRFS for HV-EOD1–4 were compared with those for LV disease using Kaplan–Meier analysis. Multivariate Cox regression analyses were conducted to investigate the prognostic value of the clinicopathological factors. In patients with BM and coexisting LM, the Kaplan–Meier method was used to compare the proportion of patients with site-specific RP at the time of castration-resistant prostate cancer (CRPC) diagnosis.

The chi-square test (or Fisher’s exact test, if appropriate) was used to compare categorical patient characteristics and the Mann–Whitney *U* test for continuous variables. All statistical analyses were conducted using Stata version 14 for Windows. All *p*-values were two-sided, and *p* < 0.05 was considered significant.

## 3. Results

### 3.1. Patient Characteristics

**Figure 1:**
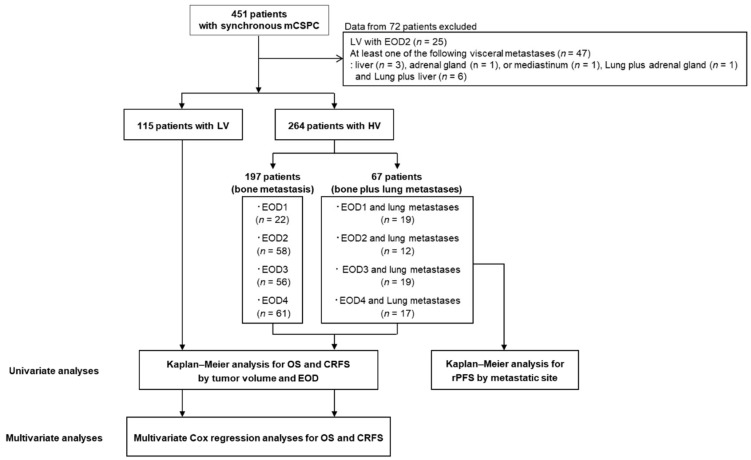
Flowchart of the study design. *mCSPC* metastatic castration-sensitive prostate cancer, *LV* low-volume, *HV-EOD1* high-volume disease with an extent of disease 1, *HV-EOD2* high-volume disease with an extent of disease 2, *HV-EOD3* high-volume disease with an extent of disease 3, *HV-EOD4* high-volume disease with an extent of disease 4, *OS* overall survival, *CRFS* castration-resistance-free survival, *RP* radiographic progression.

Among 451 patients whose data were assessed, 379 met the eligibility criteria, including 264 with HV disease and 115 with LV disease (Figure 1). Table 1 shows the patient characteristics by tumor volume and EOD; the HV group included 197 (74.6%) patients with BM alone, and 67 (25.4%) with BM and LM. The numbers of patients with HV-EOD1–4 were 41, 70, 75, and 78, respectively, and the numbers of patients with HV-EOD1–4 with LM were 19 (46.3%), 12 (17.1%), 19 (25.3%), and 17 (21.8%), respectively. All the patients with LV disease had EOD1.

Between April 2006 and September 2023, ADT or CAB were administered to 277 (73.1%) patients with mCSPC before 2018. In 2018, intensified ADT for mCSPC was approved and was subsequently administered to 76 (20.0%) patients. The remaining 26 patients (6.9%) received ADT or CAB between 2018 and September 2023 for HV-EOD4.

Regarding treatment types for CRPC in Japan, docetaxel, androgen receptor signaling inhibitor, and cabazitaxel were approved in 2008, 2014, and 2014, respectively. Of 303 patients who underwent ADT or CAB for mCSPC, 112 (37.0%) and 191 (63.0%) received therapy before 2014 and between 2014 and September 2023, respectively.

Patients with HV disease had a significantly higher initial PSA concentration, significantly greater prevalence of advanced clinicopathological features such as Gleason score (GS) ≥ 9, clinical T stage (T) ≥ 3b, clinical N1, and clinical M1a, and a significantly lower prevalence of prostate-directed radiation therapy for mCSPC compared with patients with LV disease. The proportions of patients treated with intensified ADT for mCSPC were similar across those with HV-EOD1–4 or LV disease (17.1% [*n* = 7], 24.3% [*n* = 17], 26.7% [*n* = 20], 16.7% [*n* = 13], and 16.5% [*n* = 19], respectively; *p* = 0.33) and 25.4% (*n* = 17) of patients with both BM and LM. All patients who received ADT plus docetaxel were classified as having HV-EOD4 (6.6% [*n* = 5]). No significant differences were observed in background characteristics between patients with LV and those with HV-EOD1.

Table 2 shows the clinical outcomes of CRPC and all-cause mortality by tumor volume and EOD. Patients with HV-EOD ≥ 3 had a significantly greater proportion of CRPC and all-cause mortality, compared with those who had LV or HV ≤ 2 (*p* < 0.001). The first subsequent systemic treatment with intensified ADT for CRPC accounted for approximately 75% of the patients with LV and HV-EOD1–3, with a significantly lower proportion of those with HV-EOD4 (Table S1).

### 3.2. Survival Outcomes in the Overall Cohort

**Figure 2:**
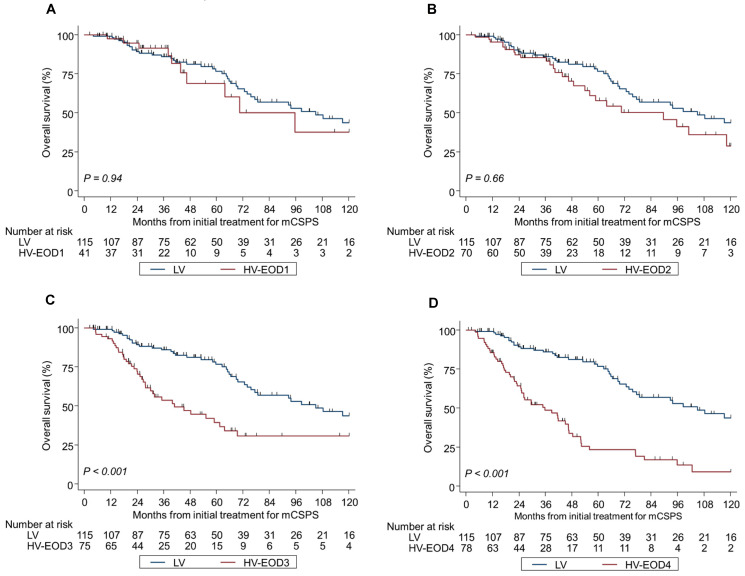
Kaplan–Meier analysis of overall survival in LV and HV-EOD1-4 (**A**–**D**). *mCSPC* metastatic castration-sensitive prostate cancer, *LV* low-volume, *HV-EOD1* high-volume disease with an extent of disease 1, *HV-EOD2* high-volume disease with an extent of disease 2, *HV-EOD3* high-volume disease with an extent of disease 3, *HV-EOD4* high-volume disease with an extent of disease 4.

**Figure 3:**
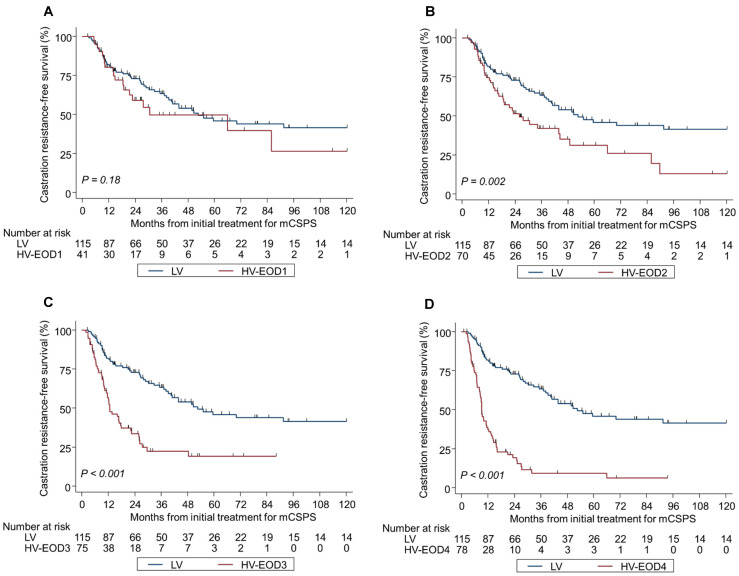
Kaplan–Meier analysis of castration resistance-free survival in LV and HV-EOD1-4 (**A**–**D**). *mCSPC* metastatic castration-sensitive prostate cancer, *LV* low-volume, *HV-EOD1* high-volume disease with an extent of disease 1, *HV-EOD2* high-volume disease with an extent of disease 2, *HV-EOD3* high-volume disease with an extent of disease 3, *HV-EOD4* high-volume disease with an extent of disease 4.

Kaplan–Meier analyses by tumor volume and EOD were significantly different in OS and CRFS during the median follow-up of 44.3 months (Figure 2A–D and Figure 3A–D); the median OS and CRFS were 105 and 52.6 months, respectively, for LV disease, and 120 and 33.8, 70.5 and 25.9, 40.9 and 12.7, and 35.8 and 9.1 months, respectively, for HV-EOD1–4 (*p* < 0.001). HV-EOD1 and HV-EOD2 showed no significant association with OS (hazard ratio [HR]: 0.97; 95% confidence interval [CI]: 0.48–1.96; *p* = 0.94, and HR: 1.26; 95% CI: 0.78–1.61; *p* = 0.66, respectively) compared with LV disease. Regarding CRFS, HV-EOD1 showed no prognostic significance (HR: 1.43; 95% CI: 0.85–2.41; *p* = 0.18) compared with LV disease, while HV-EOD2 was associated with a poor prognostic outcome (HR: 1.39; 95% CI: 1.13–1.70; *p* = 0.002). In a subgroup analysis of patients with HV-EOD2, compared with those with LV disease, survival outcomes between patients with ≤10 BM were not significantly different (median OS: 96.2 vs. 105 months; HR: 1.12; 95% CI: 0.56–2.28; *p* = 0.74; CRFS: 30.6 vs. 52.6 months; HR: 1.35; 95% CI: 0.79–2.29; *p* = 0.27) and patients with ≥11 BM had significantly worse OS (median: 59.1 months; HR: 1.87; 95% CI: 1.03–3.39; *p* = 0.040) and CRFS (median: 16.1 months; HR: 2.67; 95% CI: 1.60–4.47; *p* < 0.001) (Figure S1A,B).

### 3.3. Survival Outcomes in Patients Who Initiated mCSPC Treatment Between 2014 and September 2023

To investigate the prognostic effect of the initial treatment period on OS, 303 patients who received ADT or CAB were divided into two groups based on the 2014 approval of androgen receptor signaling inhibitors and cabazitaxel for CRPC in Japan. Patients initiating ADT or CAB before 2014 showed significantly worse OS than those starting between 2014 and September 2023 (HR: 2.84; 95% CI: 2.06–3.90; *p* < 0.001) (Figure S2). The proportion of patients starting ADT or CAB between 2014 and September 2023 was 74.0% (*n* = 71) for LV and 55.6% (*n* = 20), 58.5% (*n* = 31), 74.5% (*n* = 41), and 41.5% (*n* = 27) for HV-EOD1–4 (*p* < 0.001).

Among patients initiating mCSPC treatment between 2014 and September 2023 (*n* = 191), HRs for OS and CRFS in HV-EOD ≤ 2 were similar to those of the overall cohort, with no significant difference in the proportions of the first subsequent systemic treatment with intensified ADT among patients progressed to CRPC (LV: 75.0% [21/28], HV-EOD1: 83.3% [10/12], HV-EOD2: 83.3% [20/24], HV-EOD3: 85.2% [23/27] and HV-EOD4: 64.0% [60/25]; *p* = 0.41). HV-EOD1–2 showed no significant association with OS (median not reached; HR: 0.98; 95% CI: 0.33–2.92; *p* = 0.97, and median not reached; HR: 1.33; 95% CI: 0.89–1.98; *p* = 0.16, respectively) compared with LV disease (Figure S3A,B). Regarding CRFS, HV-EOD1 showed no prognostic significance (median: 30.0 months; HR: 1.69; 95% CI: 0.86–3.33; *p* = 0.13) compared with LV disease, whereas HV-EOD2 was associated with a poor prognostic outcome (median: 30.6 months; HR: 1.44; 95% CI: 1.07–1.93; *p* = 0.015) (Figure S4A,B). In a comparison of survival outcomes between HV-EOD ≥ 3 and LV disease, HV-EOD ≥ 3 had significantly worse OS (HV-EOD3, median: 45.2 months; HR: 1.57; 95% CI: 1.36–1.81; *p* < 0.001 and HV-EOD4, median: 41.4 months; HR: 1.46; 95% CI: 1.23–1.74; *p* < 0.001) and CRFS (HV-EOD3, median: 10.0 months; HR: 1.50; 95% CI: 1.18–1.90; *p* = 0.001 and HV-EOD4, median: 9.1 months; HR: 1.65; 95% CI: 1.37–1.99; *p* < 0.001) (Figure S3C,D and Figure S4C,D).

In HV-EOD2, survival outcomes between patients with ≤10 BM and those with LV disease had no significant difference (median OS not reached, HR: 1.37; 95% CI: 0.50–3.75; *p* = 0.54 and median CRFS: 65.9 months; HR: 1.48; 95% CI: 0.72–3.06; *p* = 0.28), whereas patients with ≥11 BM had significantly worse CRFS (median: 16.6 months; HR: 3.92; 95% CI: 1.81–8.45; *p* = 0.001). OS for patients with ≥11 BM was worse without a significant difference compared with those with LV disease (median OS not reached, HR: 1.27; 95% CI: 0.91–6.78; *p =* 0.080) (Figures S5A,B). The proportion of the first subsequent systemic treatment with intensified ADT among HV-EOD2 which progressed to CRPC was 78.5% (11/14) in ≤10 BM patients and 90.0% (9/10) in ≥11 BM *(P* = 061).

### 3.4. LM-Specific Survival Outcomes

**Figure 4:**
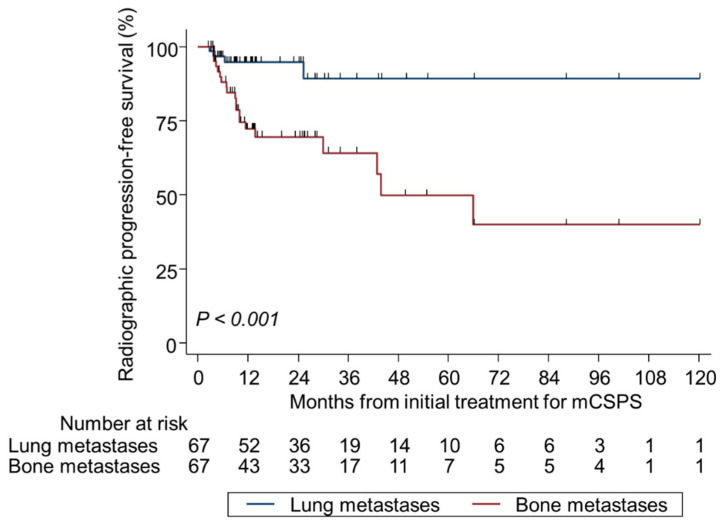
Kaplan–Meier analysis of site-specific radiographic progression-free survival at the diagnosis of castration-resistant prostate cancer in patients with bone metastasis plus lung metastasis. *mCSPC* metastatic castration-sensitive prostate cancer.

Among the 67 patients with BM and coexisting LM with a median follow-up of 31.2 months, the proportion of those with RP at CRPC diagnosis was 43.3% (*n* = 29). Regarding site-specific RP in CRPC, LM had a significantly lower incidence than BM (6.0% vs. 29.9%, *p* < 0.001) and a significantly better site-specific RP-free survival (*p* < 0.001) (Table S2, Figure 4). A new metastatic site (liver) was detected at the time of CRPC diagnosis in 7.5% of patients (*n* = 5). The number of LM did not correlate with RP to CRPC (solitary, *n* = 1, 1.5%; 2–10, *n* = 1, 1.5%; ≥ 11, *n* = 2, 3.0%; *p* = 0.62) (Table S2).

### 3.5. Multivariate Cox Regression Analyses for Survival Outcomes

Multivariate analyses for OS showed no prognostic significance for HV-EOD1 (HR 0.90; 95% CI 0.43–1.85; *p* = 0.77) or HV-EOD2 (HR 1.17; 95% CI 0.69–1.99; *p* = 0.57) compared with LV disease, whereas HV-EOD ≥ 3 was associated with a worse prognostic outcome (HR 2.03; 95% CI 1.32–3.11; *p* < 0.001) (Table 3). A similar tendency was also observed in the analyses for CRFS (HV-EOD1, HR 1.27; 95% CI 0.72–2.24; *p* = 0.40; HV-EOD2, HR 1.40; 95% CI 0.89–2.21; *p* = 0.15; HV-EOD ≥ 3, HR 4.10; 95% CI 2.72–6.78; *p* < 0.001) (Table 4). The coexistence of LM was not significantly associated with OS (HR 1.20; 95% CI 0.82–1.75; *p* = 0.35) or CRFS (HR 1.22; 95% CI 0.76–1.95; *p* = 0.41). GS ≥ 9 was an independent prognostic factor for poor OS and CRFS, while T ≥ 3b was an independent prognostic factor for poor CRFS.

## 4. Discussion

Here, we retrospectively discovered that HV disease has heterogeneous prognostic potential. Multivariate analyses showed that HV-EOD ≤ 2 was prognostically similar to LV disease, and subgroup analyses of patients initiating ADT/CAB between 2014 and September 2024 confirmed the absence of significant prognostic differences between HV-EOD1, ≤ 10 BM, and LV disease, as observed in univariate analyses of the overall cohort. Moreover, the coexistence of LM and BM did not show a significant association with OS or CRFS, regardless of the number of LM.

We assumed that subclassifying HV disease with BM would reveal various oncological characteristics, since the CHAARTED criteria define HV disease as having at least four extents of BM. Our multivariate analyses subsequently indicate that HV-EOD ≤ 2 should be prognostically distinguished from HV-EOD ≥ 3. HV-EOD ≤ 2 had no prognostic significance for OS and CRFS compared with LV disease, whereas HV-EOD ≥ 3 was significantly associated with poorer OS or CRFS. The similar survival outcomes for HV-EOD1 and LV disease are logical, given the robust evidence of a favorable prognosis for oligometastatic PCa based only on the number of metastases, regardless of the metastatic site [15]. Conversely, the prognostic value of HV-EOD2 should be cautiously considered owing to the wide range of BM (6–20) on EOD2. Previous research on EOD-based analysis discovered significantly worse OS for non-subclassified EOD2 compared to EOD1 in patients with mCSPC initially treated with ADT [11]. Here, ≤10 BM had prognoses similar to those of LV disease in the overall cohort and in patients who initiated nonupfront therapy between 2014 and 2023, indicating that EOD2 may reflect the dual biological nature of oligometastatic and widespread PCa. Recent studies have reported this dual biological nature of EOD2 using the same cutoff value [16,17]. For instance, in a multicenter cohort study of Japanese patients initially treated with ADT for mCSPC, the highest HR for OS among the five thresholds in the number of BM (≥4, ≥6, ≥11, ≥16, and ≥21) was found in the ≥ 11 BM (HR: 2.766) [16]. Therefore, HV disease should not be uniformly categorized by prognosis, and HV disease with ≤ 10 BM might be suitable candidates for an androgen receptor signaling inhibitor plus ADT, which could avoid overtreatment while adding docetaxel to doublet therapy. Currently, a clinical trial is ongoing to investigate the biomolecular profile of BM in various types of malignancies, including PCa. The results from this trial will hopefully be informative to help select the optimal treatment for HV with ≤10 BM [18].

Another important finding was that LM could be interpreted separately from the aggressive behavior of VM. The multivariate analysis indicated that LM lacks prognostic significance from two clinical perspectives. First, Kaplan–Meier analysis showed comparable survival outcomes for patients with similar backgrounds between LV and HV-EOD1, despite the latter having a prevalence of LM as high as 46%. Second, the frequency of LM with site-specific RP for CRPC was approximately five times lower than that for BM with site-specific RP. The literature has predominantly referred to the prognostic value of solitary LM; however, findings showed in some reports align with those of this study [19,20,21]. A large retrospective study based on real-world data from 16,643 patients with mCSPC in the United States showed similar 5-year survival rates between patients with BM and LM and those with BM alone [19]. Furthermore, a post hoc analysis from the LATITUDE trial discovered that the benefit of adding abiraterone to ADT in patients with LM (HR 0.60) was comparable to that in those without VM (HR 0.58) and greater than that in those with LM (HR 0.82) [20]. Subsequently, it is reasonable to speculate that some molecular mechanisms underlie these consistent results of the mildly aggressive behavior of LM. Some of the potential mechanisms for this behavior have been investigated in a limited number of basic studies. For instance, LM may share a genomic landscape similar to that of nonmetastatic PCa, as shown in a retrospective study of metachronous mCSPC in which TP53 and DNA damage repair gene mutations were absent [22]. Moreover, cluster analysis revealed that LM may have a highly immunogenic status regardless of the primary tumor origin, showing greater infiltration of myeloid dendritic cells and cytotoxic lymphocytes into cancer cells than other metastatic sites, including the liver, brain, and bone [23]. Therefore, we suggest that from the perspective of tumor volume, clinicians should focus on the number of BM when deciding the treatment intensity for mCSPC in cases involving BM and LM.

A significant limitation of using the CHAARTED criteria to define HV disease is that they do not include a GS ≥ 9 or T ≥ 3b. This multivariate analysis revealed the aggressive biology of GS ≥ 9 and T ≥ 3b. Our previous study revealed that up to 25% of patients with nonmetastatic CSPC with GS ≥ 9 and T ≥ 3b progressed to death from cancer during a median follow-up of 74 months [24]. The formidable biology associated with these two advanced characteristics may involve their androgen receptor-independent nature. The CHAARTED trial and a meta-analysis based on the STOPCAP program showed that these two advanced characteristics had predictive value for an additional OS benefit of docetaxel compared with ADT alone [4,25,26,27,28]. Therefore, the CHAARTED criteria should be subcategorized to identify the optimal treatment intensity for HV disease based on the EOD and location of the VM, and also on the presence of advanced clinical features. Given the current upfront treatment era, adding an androgen receptor signaling inhibitor to docetaxel plus ADT may help balance the benefit and adverse effects of docetaxel, especially for those with EOD ≥ 3 and at least GS ≥ 9, T ≥ 3b, or VM except for LM. In the future, a more accurate assessment of tumor volume is expected after establishing a quantitative evaluation method using prostate-specific membrane antigen positron emission tomography/computed tomography [29].

This study had some limitations. First, its retrospective design may have introduced a bias in patient selection. Second, the absence of standardized follow-up protocols such as central imaging may have affected our results. Third, most patients received non-ADT-intensified treatment for mCSPC; therefore, the results should be interpreted with caution when applied to the current era of upfront treatment. Fourth, one should be cautious when understanding the results of the subgroup analysis of HV-EOD2, given the limited number of patients with EOD2 (*n* = 70) in the univariate model. Fifth, a shorter OS observed in patients receiving nonupfront therapy initiated before 2014, compared with those starting thereafter, suggests that evolving standard of care over the long-term follow-up period may affect the results. However, the proportion of patients stratified between 2014 and September 2023 with LV disease was higher than that of those with HV-EOD ≤ 2. Moreover, the prevalence of first-sequence treatment for CRPC was similar between patients with LV disease and those with HV-EOD1–3. This enhanced the validity of the multivariate analysis results for OS. Additionally, CRFS, generally accepted as a surrogate prognostic marker for OS, relatively reflects the initial treatment effect for mHSPC. This multivariate analysis for CRFS also showed no significant difference between LV and HV-EOD ≤ 2. Sixth, the precise evaluation of the RP of LM remains challenging even by computed tomography; nevertheless, these multivariate analyses focused on the mere presence of LM before treating mCSPC, showing no additional prognostic value of LM to BM. Moreover, the small sample size of patients with BM and LM (*n* = 67) limits the generalizability of the result regarding LM’s insignificant prognostic value. However, site-specific RP to CRPC was evaluated in patients with BM and LM, enabling us to compare survival differences between BM and LM within the same clinicopathological background. Therefore, we believe our findings regarding LM are clinically informative and require further investigation.

## 5. Conclusions

This study shows that HV disease may not be categorized into a single prognostic category. Specifically, patients with EOD ≤ 2, especially those with ≤10 BM and even those with coexisting LM, may be interpreted separately from the prevalent notion of an unfavorable prognosis for HV. However, these findings are based on a small sample size including patients with EOD2 and those with LM, and the majority of the present cohort received non-upfront therapy for mCSPC. Therefore, further studies are needed to verify the clinical application of a metastatic pattern of EOD ≤ 2, especially in cases with ≤10 BM, regardless of the coexistence of LM, and to understand if these patients can be indicated for androgen receptor signaling inhibitors combined with ADT for mCSPC, while avoiding overtreatment using androgen receptor signaling inhibitors and ADT combined with docetaxel.

## Figures and Tables

**Table 1:** Patient background characteristics.

	LV (*n* = 115)	HV (*n* = 264)				***p***
		EOD1	EOD2	EOD3	EOD4	
		(*n* = 41)	(*n* = 70)	(*n* = 75)	(*n* = 78)	
Age (yr), median (range)	72 (45–92)	73 (50–85)	70 (47–84)	74 (46–91)	71 (47–81)	0.71
Charlson Comorbidity Index ≥ 1	35 (30.4)	14 (34.1)	21 (30.0)	28 (37.3)	25 (32.1)	0.86
PSA (ng/mL), median (range)	96 (5–6503)	147 (2–9747)	139 (8–1302)	400 (30–2590)	946 (2–12,409)	0.04
Gleason score ≥ 9	50 (43.5)	20 (48.8)	47 (70.0)	48 (64.0)	54 (69.2)	<0.001
Clinical T stage ≥ 3b	68 (59.1)	27 (65.9)	26 (37.1)	56 (74.7)	54 (69.2)	<0.001
Clinical N1	56 (48.6)	20 (48.8)	46 (65.7)	53 (70.7)	40 (51.3)	0.010
Metastatic status						
Lymph node metastasis	14 (12.2)	7 (17.1)	22 (31.4)	21 (28.0)	24 (30.8)	0.005
Lung metastasis	0 (0)	19 (46.3)	12 (17.1)	19 (25.3)	17 (21.8)	<0.001
Bone metastasis	115 (100)	41 (100)	70 (100)	75 (100)	78 (100)	–
Treatment type						
ADT/CAB	96 (83.5)	34 (82.9)	53 (75.7)	55 (73.3)	65 (83.3)	0.34
Intensified ADT	19 (16.5)	7 (17.1)	17 (24.3)	20 (26.7)	13 (16.7)	
PDRT	47 (40.9)	13 (31.7)	14 (20.0)	12 (16.0)	8 (10.3)	<0.001

Data are presented as *n* (%), unless stated otherwise. *LV* low-volume, *HV* high-volume, *EOD* extent of disease, *PSA* prostate-specific antigen, *ADT* androgen deprivation therapy, *CAB* combined with androgen blockade and *PDRT* prostate-directed radiation therapy.

**Table 2:** Clinical outcomes of CRPC and all-cause mortality.

	LV (*n* = 115)	HV (*n* = 264)				*p*
		EOD1	EOD2	EOD3	EOD4	
		(*n* = 41)	(*n* = 70)	(*n* = 75)	(*n* = 78)	
CRPC	51 (44.3)	20 (48.8)	42 (60.0)	52 (69.3)	65 (83.3)	<0.001
All-cause mortality	39 (33.9)	10 (24.4)	27 (38.6)	38 (50.7)	38 (48.7)	0.010

Data are presented as *n* (%) unless stated otherwise. *LV* low-volume, *HV* high-volume, *EOD* extent of disease, *CRPC* castration-resistant prostate cancer.

**Table 3:** Multivariate analyses for overall survival.

Variable	Reference	Category	HR	95% CI	*p*
Metastatic pattern	LV	HV-EOD ≥ 3	2.31	1.46–3.68	<0.001
		HV-EOD2	1.17	0.69–1.99	0.57
		HV-EOD1	0.9	0.43–1.85	0.77
Lung metastasis	Absence	Presence	1.29	0.80–2.07	0.29
Age (yr)	≤74	≥75	1.16	0.82–1.66	0.39
PSA at baseline	<100 ng/mL	≥100 ng/mL	0.75	0.50–1.11	0.15
Gleason score	≤8	≥9	1.46	1.03–2.05	0.031
Clinical T stage	≤T3a	≥T3b	1.43	0.99–2.07	0.055
Clinical M1a	Absence	Presence	1.44	0.92–2.26	0.11
Initial treatment	ADT/CAB	Intensified ADT	0.37	0.18–0.76	0.007
PDRT	No	Yes	0.79	0.58–1.09	0.16

*LV* low-volume disease, *HV* high-volume disease, *EOD* extent of disease, *HR* hazard ratio, *CI* confidence interval, *PSA* prostate-specific antigen, *ADT* androgen deprivation therapy, *CAB* combined androgen blockade, *PDRT* prostate-directed radiation therapy.

**Table 4:** Multivariate analyses for castration-resistance-free survival.

Variable	Reference	Category	HR	95% CI	*p*
Metastatic pattern	LV	HV-EOD ≥3	4.1	2.72–6.78	<0.001
		HV-EOD2	1.40	0.89–2.21	0.15
		HV-EOD1	1.27	0.72–2.24	0.41
Lung metastasis	Absence	Presence	1.23	0.85–1.80	0.28
Age (yr)	≤74	≥75	0.81	0.59–1.11	0.19
PSA at baseline	<100 ng/mL	≥100 ng/mL	1.17	0.83–1.67	0.36
Gleason score	≤8	≥9	1.43	1.06–1.92	0.018
Clinical T stage	≤T3a	≥T3b	1.78	1.28–2.46	0.001
Clinical M1a	Absence	Presence	1.11	0.71–2.21	0.15
Initial treatment	ADT/CAB	Intensified ADT	0.23	0.15–0.39	<0.001
PDRT	No	Yes	0.46	0.32–0.66	<0.001

*LV* low-volume disease, *HV* high-volume disease, *EOD* extent of disease, *HR* hazard ratio, *CI* confidence interval, *PSA* prostate-specific antigen, *ADT* androgen deprivation therapy, *CAB* combined androgen blockade, *PDRT* prostate-directed radiation therapy.

## Data Availability

Data is contained within the article or supplementary material.

## Supplementary Materials

The following supporting information can be downloaded at: https://www.mdpi.com/article/10.3390/cancers17203306/s1, Figure S1: Kaplan–Meier analysis of overall survival in LV and HV-EOD2, with a cutoff value for bone metastases of 10; Figure S2: Kaplan–Meier analysis of overall survival based on the timing of ADT or CAB initiation for mCSPC; Figure S3: Kaplan–Meier analysis of overall survival in patients who initiated treatment between 2014 and September 2023 for LV and HV-EOD1–4; Figure S4: Kaplan–Meier analysis of castration resistance-free survival in patients who initiated treatment between 2014 and September 2023 for LV and HV-EOD1–4; Figure S5: Kaplan–Meier analysis of overall survival and castration resistance-free survival in patients who initiated treatment between 2014 and September 2023 for LV, HV-EOD1, or HV-EOD2, with a cutoff value for bone metastases of 10; Table S1: First subsequent systemic treatment for patients who developed castration-resistant prostate cancer; Table S2: Detailed progression patterns of lung metastases.
