# Supplementary material for: Prognostic Value of Bone Metastases by Extent of Disease and Lung Metastases in High-Volume Castration-Sensitive Prostate Cancer: A Retrospective Study
Source: Cancers (Basel). 2025 Oct 13;17(20):3306. doi: 10.3390/cancers17203306 (PMC12563537; doi:10.3390/cancers17203306)
Supplement: Supplementary file 1 [file cancers-17-03306-s001.zip › cancers-3929567-supplementary.pdf]

Supplementary Table S1 First subsequent systemic treatment for patients who developed castration-resistant prostate cancer.

Variables, n (%)	LV (*n* = 51)	HV-EOD1 (*n* = 20)	HV-EOD2 (*n* = 42)	HV-EOD3 (*n* = 52)	HV-EDO4 (*n* = 65)	***P***
ARSI or docetaxel	37 (72.5)	15 (75.0)	32 (76.2)	39 (75.0)	31 (47.7)	0.006
ARPI or docetaxel for mCSPC	3 (5.9)	0	3 (7.1)	6 (11.5)	2 (3.1)	0.49
ADT/CAB for mCSPC	34 (66.7)	15 (75.0)	29 (69.0)	33 (63.5)	29 (44.6)	0.031
Addition of NA	2 (3.8)	0	1 (2.4)	1 (1.9)	5 (7.7)	0.55
AAT	6 (11.7)	1 (5.0)	5 (11.9)	6 (11.5)	11 (16.9)	0.76
PSL	2 (3.8)	0	1 (2.4)	0	3 (4.6)	0.60
Continuation of treatment for mCSPC	2 (3.8)	2 (10.0)	2 (4.8)	2 (3.8)	7 (10.8)	0.45
Death	2 (3.8)	2 (10.0)	1 (2.4)	4 (7.7)	8 (12.3)	0.28

LV low-volume, *HV* high-volume, *EOD* extent of disease, *ARSI* androgen receptor signaling inhibitor, *mCSPC* metastatic castration-sensitive prostate cancer, *NA* nonsteroidal antiandrogen, *AAT* alternative antiandrogen therapy, *PSL* prednisolone.

Supplementary Table S2 Detailed progression patterns of lung metastases.

**Variable**	***n* = 67**
Number of lung metastases at baseline	
1	7 (10.4)
2–10	24 (35.8)
≥11	36 (53.7)
Progression by number of lung metastases	
All	8 (11.9)
1	2 (3.0)
2–10	1 (1.5)
≥11	5 (7.5)
Progression at CRPC diagnosis by number of lung metastases	
ALL	4 (6.0)
1	1 (1.5)
2–10	1 (1.5)
≥11	2 (3.0)
Progression after CRPC diagnosis by number of lung metastases	
All	4 (6.0)
1	1 (1.5)
2–10	0
≥11	3 (4.5)
Treatment type at the progression of lung metastasis	
At CRPC diagnosis	
ADT/CAB	3 (4.5)
ARSI	1 (1.5)
After CRPC diagnosis	
ARSI	3 (4.5)
Docetaxel	1 (1.5)

CRPC castration-resistant prostate cancer, *ADT* androgen deprivation therapy, *CAB* combined androgen blockade, *ARPI* androgen receptor signaling inhibitor

Supplementary Figure S1A

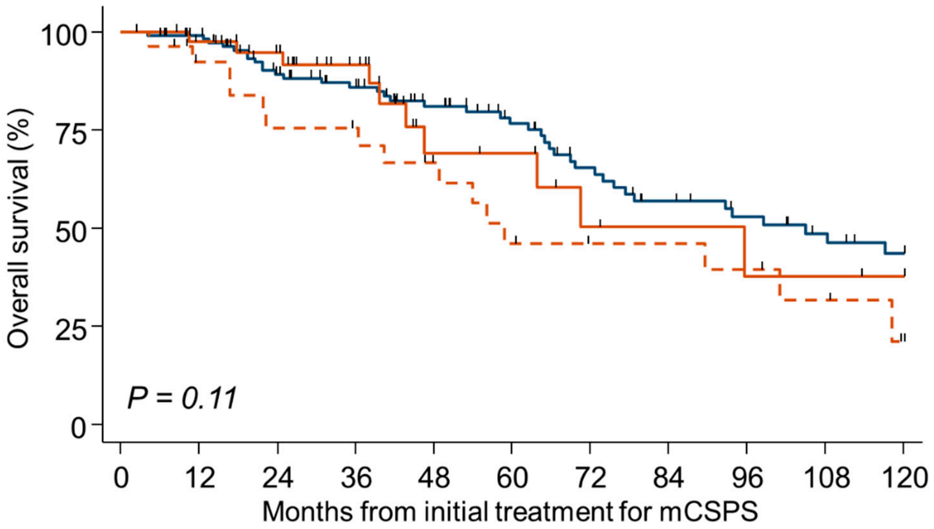

Number at risk

LV	115	107	87	75	62	50	39	31	26	21	16
≤10	41	38	32	22	10	9	5	4	3	3	2
11≤	29	22	18	17	13	9	7	7	6	4	1

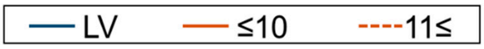

Supplementary Figure S1B

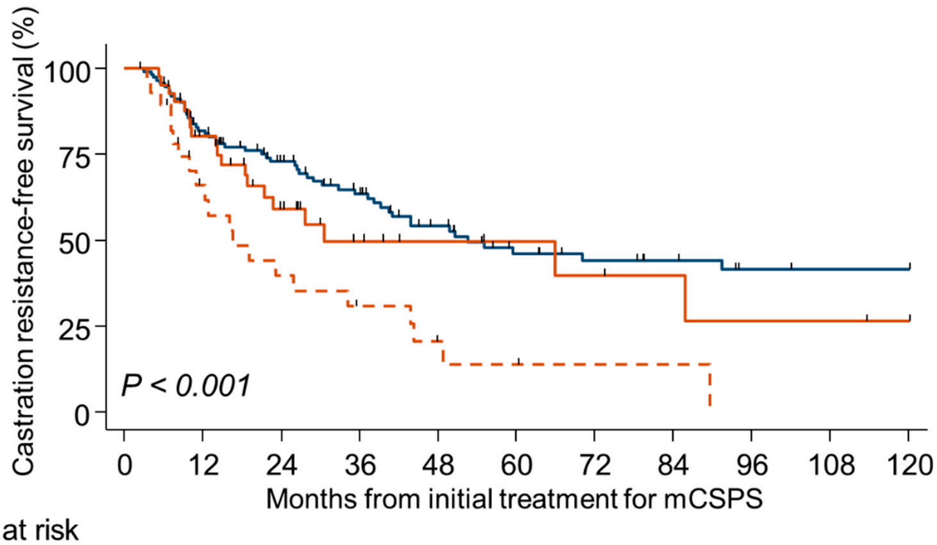

Number at risk

LV	115	87	66	50	37	26	22	19	15	14	14
≤10	41	30	17	9	6	5	4	3	2	2	1
11≤	29	15	9	6	3	2	1	1	0	0	0

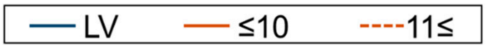

Supplementary Figure S2

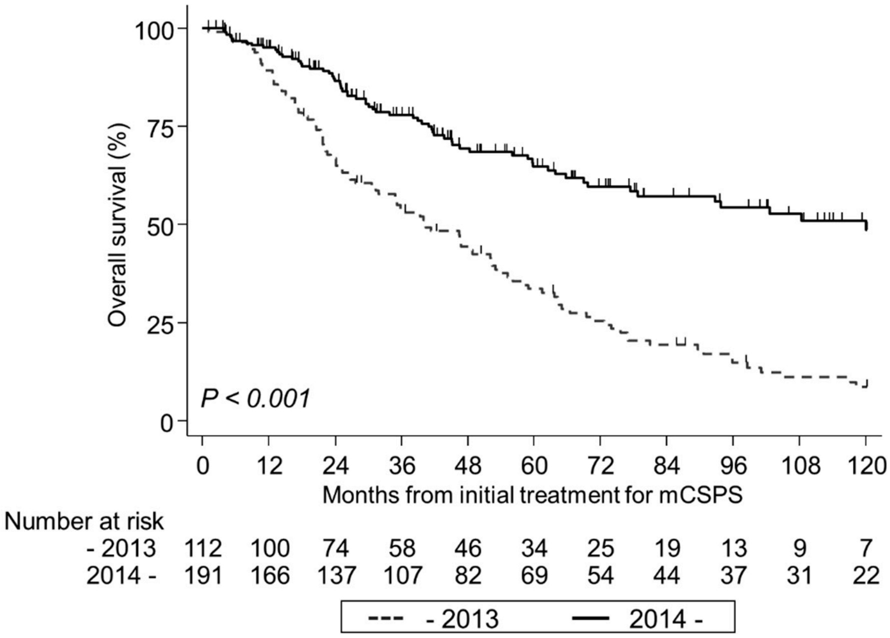

Supplementary Figure S3

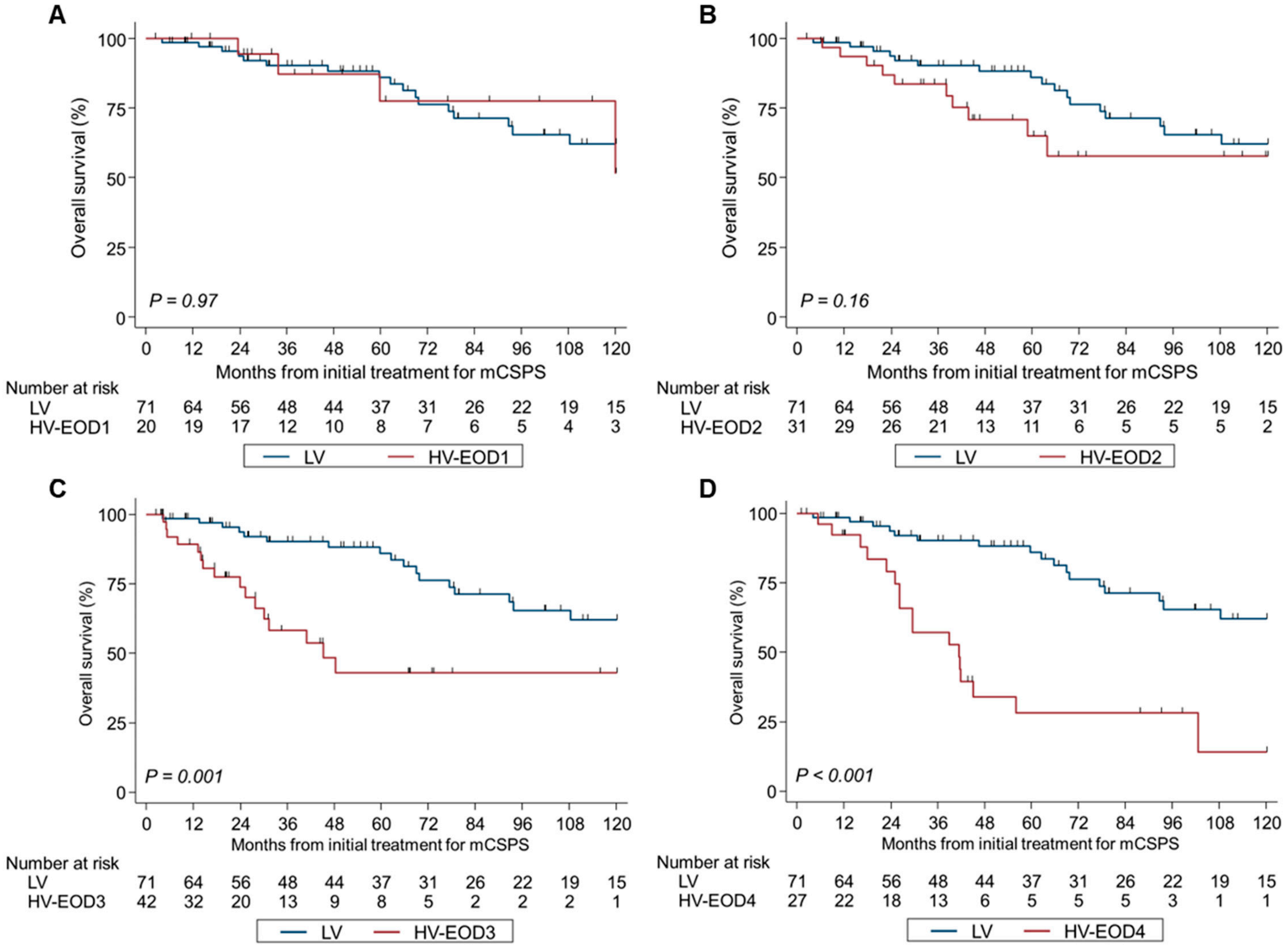

Supplementary Figure S4

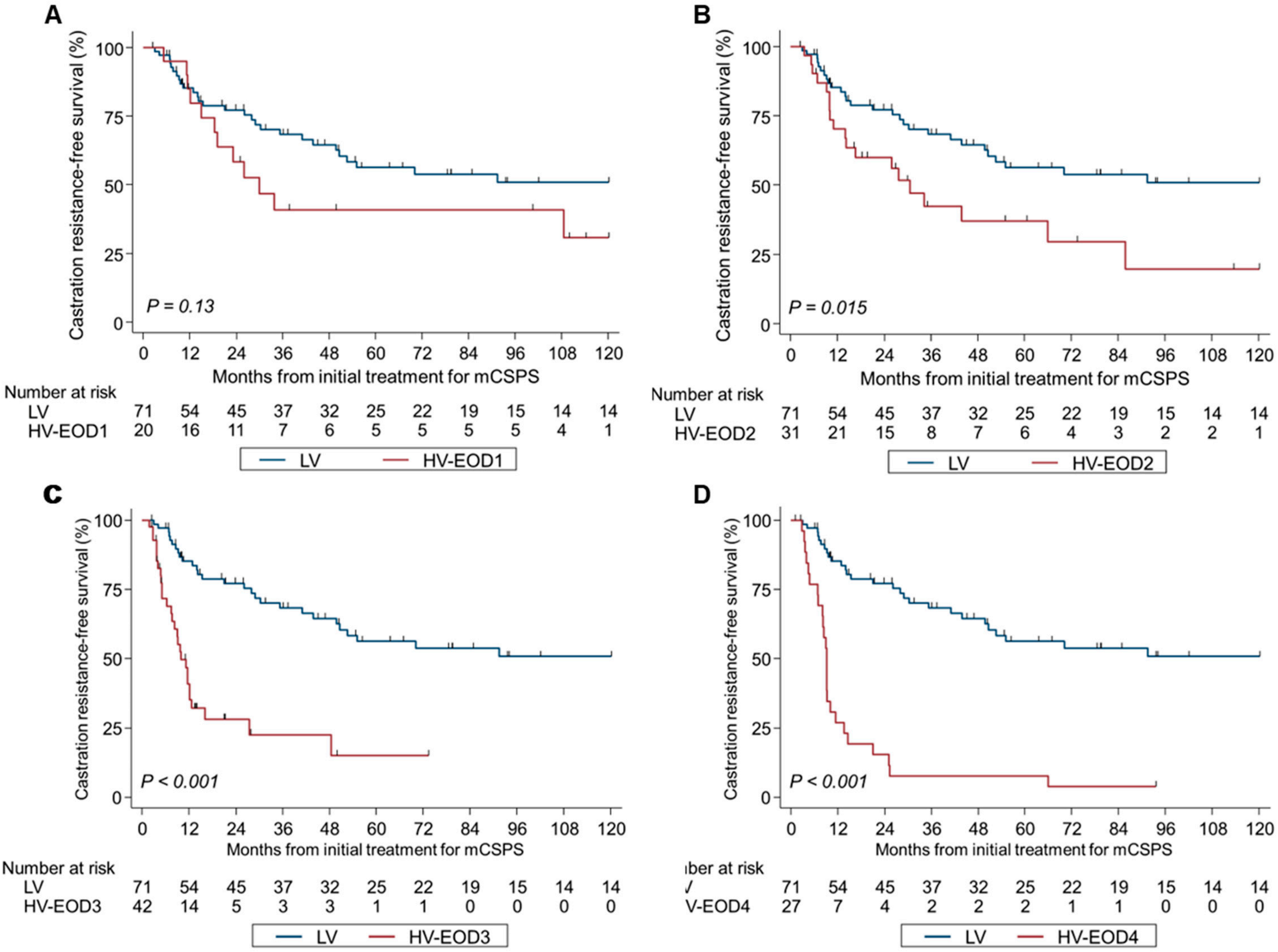

Supplementary Figure S5A

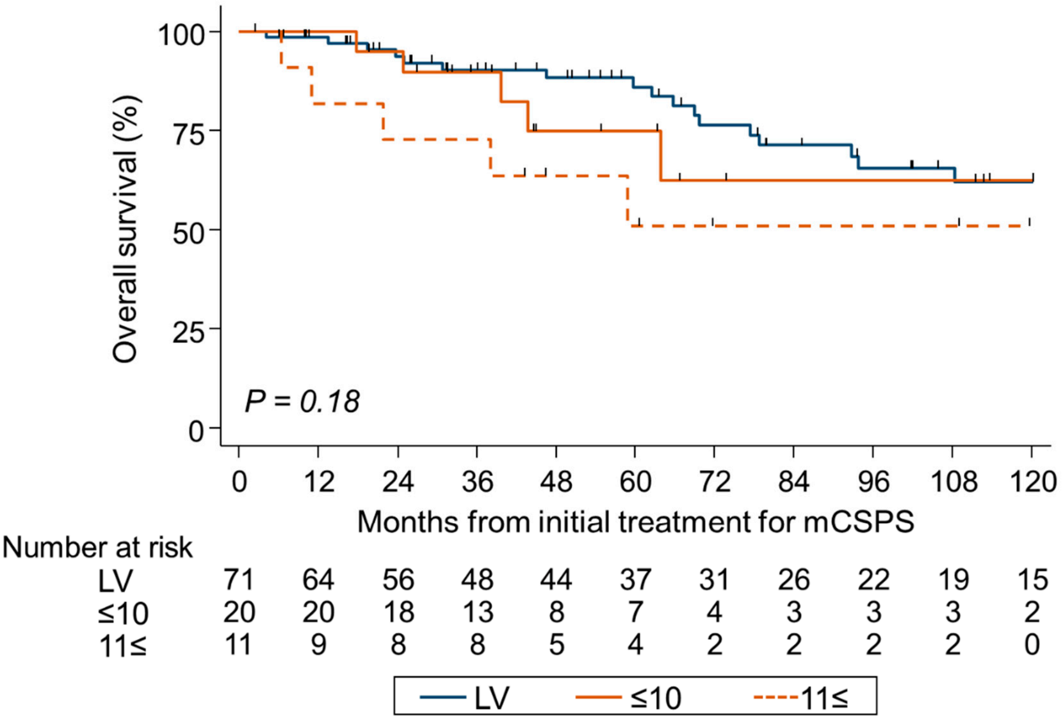

Supplementary Figure S5B

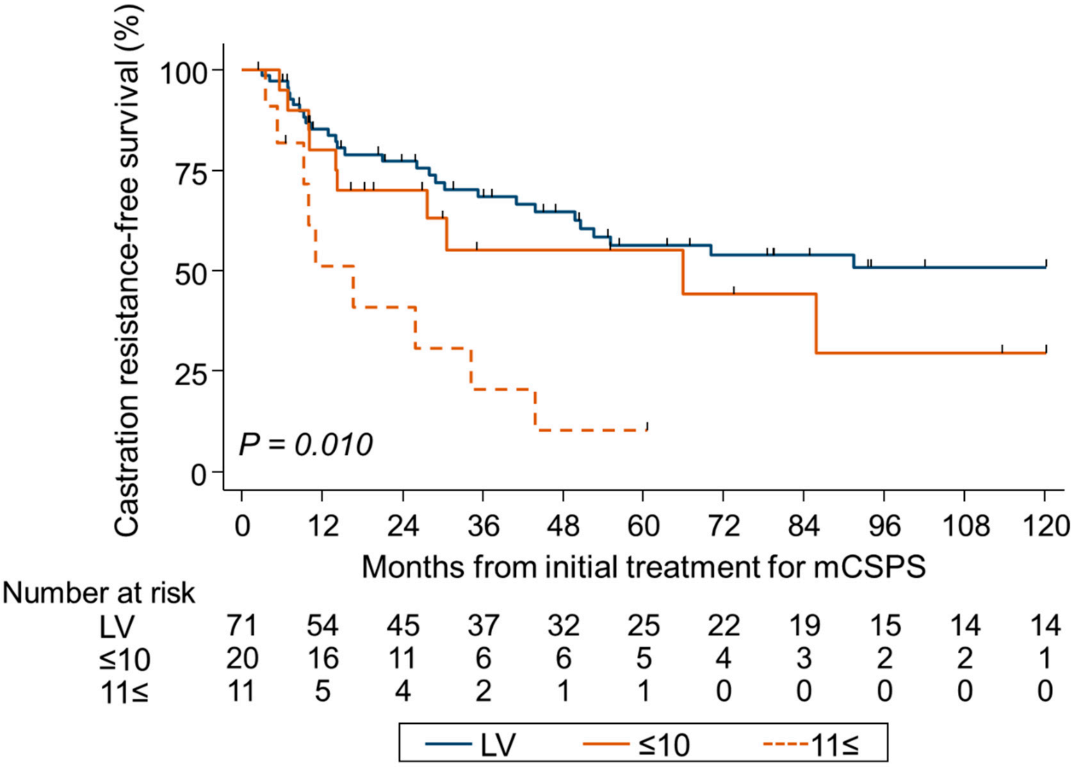

Supplementary Figure S1

Kaplan–Meier analysis of overall survival (A) and castration resistance-free survival (B) in LV and HV-EOD2 with a cut-off value of bone metastases of 10.

mCSPC metastatic castration-sensitive prostate cancer, *LV* low-volume, *HV-EOD2* high-volume disease with an extent of disease 2

Supplementary Figure S2

Kaplan–Meier analysis of overall survival based on the timing of the initiation of ADT or CAB for mCSPC.

ADT androgen deprivation therapy, *CAB* combined androgen blockade, *mCSPC* metastatic castration-sensitive prostate cancer.

Supplementary Figure S3

Kaplan–Meier analysis of overall survival in patients who initiated treatment between 2014 and September 2023 for LV and HV-EOD1-4 (A-D).

mCSPC metastatic castration-sensitive prostate cancer, *LV* low-volume, *HV-EOD1* high-volume disease with an extent of disease 1, *HV-EOD2* high-volume disease with an extent of disease 2, *HV-EOD3* high-volume disease with an extent of disease 3, *HV-EOD4* high-volume disease with an extent of disease 4.

Supplementary Figure S4

Kaplan–Meier analysis of castration resistance-free survival in patients who initiated treatment between 2014 and September 2023 for LV and HV-EOD1-4 (A-D).

mCSPC metastatic castration-sensitive prostate cancer, *LV* low-volume, *HV-EOD1* high-volume disease with an extent of disease 1, *HV-EOD2* high-volume disease with an extent of disease 2, *HV-EOD3* high-volume disease with an extent of disease 3, *HV-EOD4* high-volume disease with an extent of disease 4.

Supplementary Figure S5

Kaplan–Meier analysis of overall survival (A) and castration resistance-free survival (B) in patients who initiated treatment between 2014 and September 2023 for LV, HV-EOD1 or HV-EOD2 with a cut-off value of bone metastases of 10.

mCSPC metastatic castration-sensitive prostate cancer, *LV* low-volume, *HV-EOD2* high-volume disease with an extent of disease 2
